# Suprachoroidal Triamcinolone Acetonide for the Treatment of Refractory Macular Edema Secondary to Non-Infectious Uveitis

**DOI:** 10.21203/rs.3.rs-7787490/v1

**Published:** 2025-10-26

**Authors:** Menke Bryant, Charlene Choo, Marc Ohlhausen, Nam Nguyen, Lindsay Helget, Alan Erickson, Christopher Conrady, Steven Yeh

**Affiliations:** University of Miami; University of Nebraska Medical Center; University of Nebraska Medical Center; University of Nebraska Medical Center; University of Nebraska Medical Center; University of Nebraska Medical Center; University of Nebraska Medical Center; University of Nebraska Medical Center

**Keywords:** suprachoroidal triamcinolone acetonide, uveitis, macular edema

## Abstract

**Introduction:**

Suprachoroidal triamcinolone acetonide (TA) was recently FDA-approved and is emerging as a new alternative to other local therapies for macular edema (ME) associated with noninfectious uveitis (NIU).

**Methods:**

This was a retrospective review of our initial experience with a cohort of patients with refractory ME secondary to NIU treated with suprachoroidal TA from November 2022 to October 2023. Data collected included demographics, ophthalmic history, as well as exam and imaging findings at baseline and follow-up visits.

**Results:**

Six eyes from 5 patients with refractory ME secondary to NIU were included in the study. The cohort included 2 females (40%) and the median age was 62 years (IQR = 8). Ophthalmic diagnoses included intermediate uveitis (n = 2; 40%), birdshot chorioretinopathy (n = 1; 20%), autoimmune retinopathy (n = 1; 20%), and panuveitis (n = 1; 20%). The median logMAR visual acuity was 0.7 (Snellen 20/100) at baseline and improved to 0.3 (Snellen 20/40) during follow-up visits at 1 month and 2–3 months. The median central subfield thickness (CST) was 690 μm at baseline and improved to 367.5 μm and 309 μm at the follow-up visits at 1 month and 2–3 months, respectively. The initial improvement in logMAR visual acuity and CST was less pronounced at follow-up visits at 6–7 months and 11–12 months.

**Conclusions:**

This study demonstrates the safety of suprachoroidal TA and efficacy signals including improvement in visual acuity and ME at 3 months in patients with severe, refractory ME secondary to NIU.

## Introduction

Macular edema (ME) is the most common complication of uveitis and a major cause of visual impairment in patients with chronic uveitis.^1,2^ Systemic and locally administered corticosteroids are the mainstay of treatment for noninfectious uveitis (NIU) complicated by ME, but a recent survey study of uveitis and retina specialists found that local therapy was preferred for unilateral or posterior segment disease.^3^ Commonly used local therapies included intravitreal dexamethasone implant (Ozurdex^®^), periocular triamcinolone acetonide (TA), and intravitreal TA. In the Multicenter Uveitis Steroid Treatment (MUST) clinical trial, patients with intermediate, posterior, and panuveitis who were treated with sustained-release fluocinolone acetonide (FA) implants had comparable visual outcomes to those treated with systemic corticosteroids.^4^ However, local corticosteroid implant was associated with a higher risk for elevated intraocular pressure (IOP) and cataract surgery. This was followed by the PeriOcular versus INTravitreal corticosteroids for uveitis macular edema (POINT) trial that compared the safety and efficacy of various forms of local corticosteroid therapy.^5^ Results of the POINT study showed that intravitreal TA and dexamethasone implants were superior to periocular TA in improving or resolving ME associated with uveitis but were associated with a higher risk of elevated IOP.

Suprachoroidal injection is a novel local drug delivery method that targets the potential space between the sclera and choroid. The favorable pharmacokinetics include higher levels of the therapeutic agent within posterior segment layers, including the choroid and retina, and lower levels of the agent in anterior segment structures with the potential for greater efficacy and reduced adverse events including cataract and IOP.^6^ The favorable pharmacokinetic profile of suprachoroidal drug delivery has prompted investigations for its use in various diseases affecting the posterior segment, including ME secondary to diabetics, retinal vein occlusion, and uveitis, age-related macular degeneration, and choroidal melanoma.^7^ Recent clinical trials have demonstrated the safety and efficacy of suprachoroidal injection of TA suspension in patients with ME secondary to NIU over a period of up to 48 weeks. In 2021, suprachoroidal injection of TA suspension CLS-TA (Xipere^™^) was FDA-approved for the treatment of ME associated with uveitis.^8^

In this study, we evaluated the safety and efficacy of suprachoroidal TA in a limited cohort of patients with refractory ME secondary to NIU. Our results show favorable vision and OCT outcomes during short-term follow-up with potential need for adjunctive agents over time in patients with more refractory inflammatory eye disease.

## Methods

This study was a retrospective review of patients with refractory ME secondary to NIU. Patients who demonstrated recurrent macular edema after one or more local or systemic therapies who were treated with suprachoroidal corticosteroid injections from 2021 to 2023 at the Truhlsen Eye Institute, University of Nebraska Medical Center (UNMC) were included. Approval for this study was obtained from the Institutional Review Board at UNMC. Patients were treated according to the best medical judgement of providers following standard-of-care practices with appropriate consultations from other services as clinically indicated. The Standardization of Uveitis Nomenclature was used to classify the anatomic location of uveitis, including anterior, intermediate, posterior, and panuveitis.^9^

### Data collection

Data were collected on demographic information, ophthalmic history, as well as exam, imaging findings at baseline visit and during follow-up within 1 month, at 2–3 months, 6–7 months, and 11–12 months. Optical coherence tomography (OCT) scans were reviewed for central subfield thickness (CST), defined as the central 1-mm zone with automated segmentation software on the Zeiss Cirrus 5000^™^ spectral-domain OCT. CST was measured with calipers in images with segmentation errors. For adverse events following suprachoroidal injections, data were collected on IOP and cataract development in addition to serious adverse events, such as retinal detachment, endophthalmitis, or serious illness requiring hospitalization.

### Statistical analysis

Snellen visual acuities (VA) were converted to logarithm of the minimum angle of resolution (logMAR) values.^10^ Descriptive and inferential statistical analyses were performed with median VA reported along with the interquartile ranges (IQR) at the designated follow-up time points. Wilcoxon signed-rank test was used to compare the median logMAR VA, CST, and IOP from baseline to each follow-up visit. Eyes with light perception vision or worse were excluded from analysis (n = 1).

## Results

This study included five patients with refractory ME secondary to NIU who were treated with suprachoroidal TA (Table 1). The cohort had a median age of 62 years (IQR = 8) and included 2 females (40%). All patients were white or Caucasian (100%). The ophthalmic diagnoses included intermediate uveitis (n = 2; 40%), birdshot chorioretinopathy with positive HLA-A29 (n = 1; 20%), autoimmune retinopathy (n = 1; 20%), and panuveitis (n = 1; 20%). The most common previous systemic treatment included oral prednisone (n = 3; 60%) and methotrexate (n = 2; 40%). Two patients (40%) were on oral prednisone at the time of suprachoroidal TA administration. One of these patients was also on methotrexate (20%) and adalimumab (20%). One patient was on cyclosporine (20%) at the time of suprachoroidal TA administration.

Six eyes from 5 patients with refractory ME secondary NIU treated were treated with suprachoroidal TA (Table 2). Four out of 6 eyes were pseudophakic (66.7%). At the baseline visit, the median logMAR VA was 0.7 (IQR = 0.17) and median CST was 690 μm (IQR = 568). The most common previous local treatment was intravitreal steroid implant (n = 5; 83.3%), followed by topical corticosteroids (n = 3; 50.0%) and periocular triamcinolone acetonide injection (n = 3; 50%). One patient had a previous suprachoroidal TA injection within the last two months. At the time of suprachoroidal TA injection, 2 eyes (40%) from 2 patients were being treated with topical corticosteroids.

**Figure 1:**
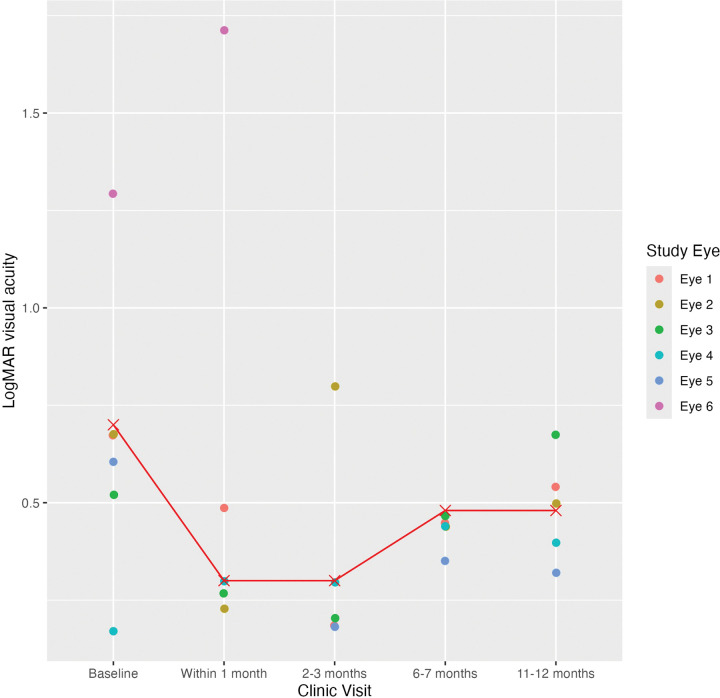
LogMAR Visual Acuity of Eyes at Baseline and Follow-up Visits Scatter plot includes logMAR visual acuity of each eye treated with suprachoroidal triamcinolone acetonide at baseline and follow-up visits. Median logMAR visual acuity at baseline and each follow-up visit is depicted by red cross mark connected by a line graph.

**Figure 2:**
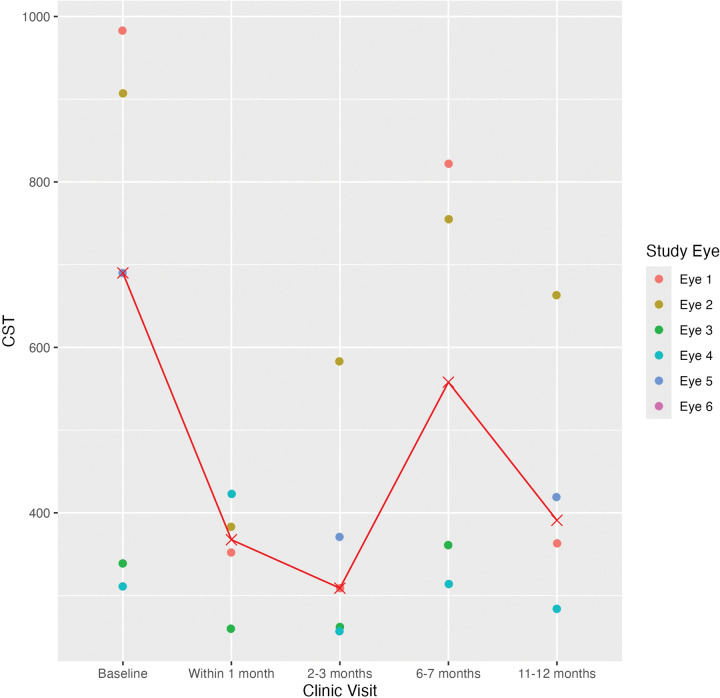
Central Subfield Thickness of Eyes at Baseline and Follo-up Visits Scatter plot includes central subfield thickness (CST) of each eye treated with suprachoroidal triamcinolone acetonide at baseline and follow-up visits. Median CST at baseline and each follow-up visit is depicted by red cross mark connect by a line graph.

**Figure 3:**
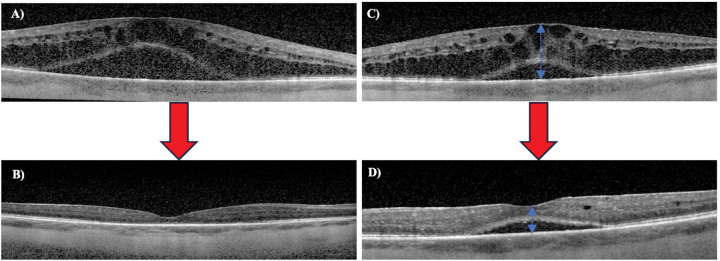
An example of the resolution of macular edema following suprachoroidal triamcinolone acetonide (TA) injection. Optical coherence tomography (OCT) image of the right eye (A) and left eye (C) showing intraretinal and subretinal fluid prior to suprachoroidal TA injection. Blue arrows represent an example measurement of central subfield thickness. B) OCT of right eye with resolution of intraretinal and subretinal fluid 7 weeks after suprachoroidal TA injection. D) OCT of the left eye with significant improvement of intraretinal and subretinal fluid 5 weeks after suprachoroidal TA injection.

The median logMAR VA was 0.7 (IQR = 0.165) at the baseline visit, 0.3 (IQR = 0.24) at the follow-up within 1 month, 0.3 (IQR = 0.12) at 2–3 months, 0.48 (IQR = 0.14) at 6–7 months, and 0.48 (IQR = 0.18) at 11–12 months (Table 3; Fig. 1). The median CST was 690 μm (IQR = 568) at the baseline visit, 367.5 μm (IQR = 64) at the follow-up within 1 month, 309 μm (IQR = 109) at 2–3 months, 771.8 μm (IQR = 422.5) at 6–7 months, and 391 μm (IQR = 136.8) at 11–12 months (Figs. 2 and 3). The median IOP was 14 mmHg (IQR = 11) at the baseline visit, 11 mmHg (IQR = 6) at the follow-up within 1 month, 14.5 mmHg (IQR = 11.8) at 2–3 months, 11 mmHg (IQR = 18) at 6–7 months, and 14.5 mmHg (IQR = 5) at 11–12 months. VA improved by 2 or more lines in 2 out of 5 eyes (40.0%) and remained stable in 3 out of 5 eyes (60.0%) at the follow-up within 1 month and 2–3 months. Four out of 5 eyes (80%) showed stable VA or improvement of 2 or more lines at 11–12 month follow-up, while one patient demonstrated vision loss from Snellen 20/100 at baseline to 20/200 at this follow-up time point. There was no statistically significant difference between the median logMAR VA and CST at the baseline and follow-up visits (p > 0.05).

The median IOP was not significantly elevated during the follow-up period compared to the baseline visit (p > 0.05). Two out of 6 phakic eyes (33.3%) did not show evidence of worsening cataracts after suprachoroidal TA injection. There were no serious adverse events, including retinal detachment, endophthalmitis, or serious illness requiring hospitalization.

## Discussion

In this small cohort of patients with refractory ME secondary to NIU, the median logMAR VA and CST improved at follow-up visits within 1 month and 2–3 months after administration of suprachoroidal TA. While the improvement in VA and OCT outcomes were notable in several patients, statistical analyses of the improvement in both measures were likely limited by the small sample size. In addition, a ceiling effect for improvement in VA may also be observed in patients with severe disease, given that concomitant improvement in CST was observed when a clear VA benefit was not observed. The trends in CST were similar to the findings observed in the PEACHTREE phase III clinical trial that compared the efficacy of suprachoroidal TA injection to a sham procedure in patients with ME secondary to NIU.^11^ Specifically, improvement in OCT outcomes were observed at 1-, 2-, and 3-months follow-up, at which time a second suprachoroidal injection was administered in the PEACHTREE trial. The PEACHTREE trial results showed that the median time to rescue therapy was 89 days in the treatment arm compared to 36 days in the control arm, suggesting that the effects of suprachoroidal TA may last at least 12 weeks. A recent retrospective study by Panse and colleagues that investigated the effect of suprachoroidal TA in 51 patients with NIU also found that almost 50% of eyes required additional treatment for ME at the 12-week follow-up.^12^ Of note, within the MAGNOLIA study, which evaluated the need for rescue therapy in a subset of patients from PEACHTREE who did not require rescue therapy, 50% of patients did not require rescue therapy at the 48-week follow-up visit, approximately 9 months after the second suprachoroidal injection in the PEACHTREE study.^13^

Our retrospective data showed reduced CST until the follow-up at 2–3 months but some recurrence of ME during follow-up visits at 6–7 months and 11–12 months. An encouraging finding was that IOP was not significantly different between the baseline and follow-up visits after administration of suprachoroidal TA. Further studies are needed to evaluate the effect of suprachoroidal TA in patients with refractory ME secondary to NIU and long-term adverse events associated with repeat treatment.

In addition to suprachoroidal TA treatment, some patients were concurrently treated with immunomodulatory therapies of varying targets of action, including anti-metabolites (methotrexate), T-cell calcineurin inhibitors (cyclosporine), and tumor necrosis factor-alpha inhibitors (adalimumab). These patients required laboratory monitoring and were jointly managed with colleagues in Rheumatology, highlighting the importance of multidisciplinary care paradigms and targeting both systemic and local inflammation in the care of complex uveitis patients.

Limitations included the retrospective nature of the study in a limited cohort of patients with refractory ME at a tertiary care, university-based institution. Patients had follow-up visits as clinically indicated, so data were not collected according to a study protocol.

Despite these limitations, our initial experience with this cohort of patients supports the safety and efficacy of suprachoroidal TA for the treatment of refractory ME associated with NIU with benefits to VA and anatomic OCT outcomes lasting up to 12 weeks. Whether scheduled injections (i.e., 2 injections scheduled at day 0 and week 12 per the PEACHTREE protocol) may lead to a greater efficacy and durability signals than a single injection protocol is an important consideration given the PEACHTREE protocol that mandated suprachoroidal injections at baseline at 3-month follow-up.^11^ Further studies regarding the optimal dosing intervals for patients with severe or refractory ME associated with NIU are needed, as well as optimal treatment paradigms to manage systemic and local medications in the care of patients with complex inflammatory eye disease.

## Figures and Tables

**Table 1:** Demographic and clinical characteristics of patients with refractory ME secondary to NIU treated with suprachoroidal triamcinolone acetonide

Characteristics	Total (n = 5)
Median age, years (IQR)	62 (8)
Female, n (%)	2 (40)
White or Caucasian race, n (%)	5 (100)
Ophthalmic diagnosis, n (%)	
Intermediate uveitis	2 (40)
Panuveitis	1 (20)
Birdshot chorioretinopathy, HLA-A29+	1 (20)
Autoimmune retinopathy	1 (20)
All previous systemic treatment, n (%)	
Oral prednisone	3 (60)
Immunomodulatory therapy	2 (40)
Methotrexate	2 (40)
Adalimumab	1 (20)
Azathioprine	1 (20)
Cyclosporine	1 (20)
Mycophenolate mofetil	1 (20)
Antiviral	1 (20)
Concurrent systemic treatment, n (%)	
Oral prednisone	2 (40)
Immunomodulatory therapy	2 (50)
Adalimumab	1 (20)
Methotrexate	1 (20)
Cyclosporine	1 (20)

ME = macular edema, NIU = noninfectious uveitis, n = number of patients, IQR = interquartile range, HLA = human leukocyte antigen

**Table 2:** Clinical characteristics of eyes with refractory ME associated with NIU treated with suprachoroidal triamcinolone acetonide

Characteristics	Total (n = 6)
Pseudophakia, n (%)	4 (66.7)
Baseline logMAR visual acuity, median (IQR)	0.7 (0.17)
Baseline CST in pm, median (IQR)	690 (568)
Baseline intraocular pressure in mmHg, median (IQR)	14 (11)
All previous local treatment, n (%)	
Topical corticosteroid	3 (50.0)
Subtenon Kenalog	3 (50.0)
Intravitreal steroid implant	5 (83.3)
Intravitreal anti-VEGF	2 (33.3)
Suprachoroidal TA	1 (16.7)
Concurrent local treatment, n (%)	
Topical corticosteroid	2 (66.7)

ME = macular edema, NIU = noninfectious uveitis, n = number of eyes, logMAR = Logarithm of the Minimum Angle of Resolution, IQR = interquartile range, CST = central subfield thickness, anti-VEGF = anti-vascular endothelial growth factor, TA = triamcinolone acetonide

* Systemic immunosuppressive therapy was co-managed with the Division of Rheumatology at UNMC

**Table 3:** LogMAR visual acuity, CST, and IOP of eyes at baseline and follow-up visits

Clinic visit	LogMAR VA, median (IQR)	CST in μm, median (IQR)	IOP in mmHg, median (IQR)
Baseline visit	*N = 6* 0.7 (0.165)	*N = 5* 690 (568)	*N = 6* 14 (11)
Follow-up within 1 month	*N = 5* 0.3 (0.24)	*N = 4* 367.5 (64)	*N = 5* 11 *(6)*
Follow-up at 2–3 months	*N = 5* 0.3 (0.12)	*N = 5* 309 (109)	*N = 5* 14.5 (11.8)
Follow-up at 6–7 months	*N = 5* 0.48 (0.14)	*N = 4* 771.8 (422.5)	*N = 5* 11 (18)
Follow-up at 11–12 months	*N = 5* 0.48 (0.18)	*N = 4* 391 (136.8)	*N = 5* 14.5 (5)

LogMAR = Logarithm of the Minimum Angle of Resolution, CST = central subfield thickness, IOP = intraocular pressure, VA = visual acuity, IQR = interquartile range, n = number of eyes

## References

[1] Rothova A, Suttorp-van Schulten MS, Frits Treffers W, Causes and frequency of blindness in patients with intraocular inflammatory disease. Br J Ophthalmol. 1996;80(4):332–6. 10.1136/bjo.80.4.332. 8703885 PMC505460

[2] Lardenoye CWTA, van Kooij B, Rothova A. Impact of Macular Edema on Visual Acuity in Uveitis. Ophthalmology. 2006;113(8):1446–9. 10.1016/j.ophtha.2006.03.027. 16877081

[3] McHarg M, Young L, Kesav N, Practice patterns regarding regional corticosteroid treatment in noninfectious Uveitis: a survey study. J Ophthalmic Inflamm Infect. 2022;12:3. 10.1186/s12348-021-00281-z. 34982279 PMC8727651

[4] Kempen JH, Altaweel MM, Holbrook JT, Randomized Comparison of Systemic Anti-inflammatory Therapy Versus Fluocinolone Acetonide Implant for Intermediate, Posterior and Panuveitis: The Multicenter Uveitis Steroid Treatment Trial. Ophthalmology. 2011;118(10):1916–26. 10.1016/j.ophtha.2011.07.027. 21840602 PMC3191365

[5] Thorne JE, Sugar EA, Holbrook JT, Periocular triamcinolone versus intravitreal triamcinolone versus intravitreal dexamethasone implant for the treatment of uveitic macular edema: The PeriOcular versus INTravitreal corticosteroids for uveitic macular edema (POINT) Trial. Ophthalmology. 2019;126(2):283–95. 10.1016/j.ophtha.2018.08.021. 30269924 PMC6348060

[6] Patel SR, Berezovsky DE, McCarey BE, Targeted administration into the suprachoroidal space using a microneedle for drug delivery to the posterior segment of the eye. Invest Ophthalmol Vis Sci. 2012;53(8):4433–41. 10.1167/iovs.12-9872. 22669719 PMC3394664

[7] Huang Y, Choo C, Hancock S, Suprachoroidal Drug Delivery for Macular Edema Associated With Noninfectious Uveitis. J Vitreoretin Dis. 2024;8(4):401–9. 10.1177/24741264241246314. 39148567 PMC11323513

[8] Joszt L. Xipere for Macular Edema Associated With Uveitis Launches in United States. AJMC. February 16, 2022. Accessed June 14, 2023. https://www.ajmc.com/view/xipere-for-macular-edema-associated-with-uveitis-launches-in-united-states

[9] Jabs DA, Nussenblatt RB, Rosenbaum JT Standardization of uveitis nomenclature for reporting clinical data. Results of the First International Workshop. Am J Ophthalmol. 2005;140(3):509–516. 10.1016/j.ajo.2005.03.057 16196117 PMC8935739

[10] Holladay JT. Proper method for calculating average visual acuity. J Refract Surg Thorofare NJ 1995. 1997;13(4):388–91. 10.3928/1081-597X-19970701-16. 9268940

[11] Yeh S, Khurana RN, Shah M, Efficacy and Safety of Suprachoroidal CLS-TA for Macular Edema Secondary to Noninfectious Uveitis. Ophthalmology. 2020;127(7):948–55. 10.1016/j.ophtha.2020.01.006. 32173113

[12] Panse K, Hang A, Ruiz J, Suprachoroidal Triamcinolone Acetonide for Noninfectious Uveitis: Real-World Impact on Clinical Outcomes. Am J Ophthalmol. 2025;271:259–67. 10.1016/j.ajo.2024.11.022. 39645179

[13] Khurana RN, Merrill P, Yeh S Extension study of the safety and efficacy of CLS-TA for treatment of macular oedema associated with non-infectious uveitis (MAGNOLIA). Br J Ophthalmol. Published online March 12, 2021:bjophthalmol–2020. 10.1136/bjophthalmol-2020-317560 PMC9340030 33712478

